# Tricuspid annuloplasty in ischemic cardiomyopathy patients undergoing restrictive mitral annuloplasty

**DOI:** 10.3389/fcvm.2025.1542619

**Published:** 2025-05-14

**Authors:** Yusuke Misumi, Satoshi Kainuma, Daisuke Yoshioka, Takuji Kawamura, Ai Kawamura, Shin Yajima, Shunsuke Saito, Takashi Yamauchi, Masaki Taira, Kazuo Shimamura, Shigeru Miyagawa

**Affiliations:** Department of Cardiovascular Surgery, Osaka University Graduate School of Medicine, Osaka, Japan

**Keywords:** ischemic cardiomyopathy, restrictive mitral annuloplasty, tricuspid annuloplasty, functional mitral regurgitation, tricuspid regurgitation

## Abstract

**Background:**

We elucidated the impact of concomitant tricuspid annuloplasty (TAP) on postoperative tricuspid regurgitation (TR), pulmonary hypertension (PH) and survival in patients with ischemic cardiomyopathy undergoing restrictive mitral annuloplasty (RMA).

**Methods:**

This study included 234 patients with ischemic cardiomyopathy (LV ejection fraction ≤40%) who underwent RMA. Of them, 114 (49%) underwent concomitant TAP for secondary TR. The primary endpoint was freedom from significant recurrence (i.e., moderate or greater) and progression (≥2+ grades) in TR. The secondary endpoints were postoperative pulmonary artery systolic pressure (sPAP) and overall survival.

**Results:**

The 30-day mortality was not different (0.9% vs. 0.8%, *P* = 0.97), despite higher EuroSCORE II score (median, 9.3% vs. 7.2%, *P* = 0.016) for TAP group. At baseline, TAP group had higher TR grades (2.4 ± 0.8 vs. 1.4 ± 0.6, *P* < 0.001) and sPAP (51 ± 16 vs. 44 ± 12 mmHg, *P* < 0.001). At 5-year post-surgery, RMA with TAP demonstrated higher freedom from recurrence or progression of TR (91 ± 3% vs. 81 ± 4%, log-rank *P* = 0.036), yielding nearly identical sPAP (42 ± 18 vs. 40 ± 16 mmHg, *P* = 0.54). Multivariable analysis demonstrated concomitant TAP was independently associated with freedom from significant recurrence in TR. Overall survival were not different between the groups (*P* = 0.74).

**Conclusions:**

In patients with ischemic cardiomyopathy, concomitant TAP did not increase operative mortality and better reduced TR, resulting in comparable PH severity and long-term survival, compared to RMA alone.

## Introduction

Tricuspid regurgitation (TR) secondary to left-side valve disease is a known risk factor for survival and functional status (1–4). Secondary TR develops as a consequence of the pathological pathway starting from left-side valve disease via increased left atrial pressure, secondary pulmonary hypertension (PH), and right ventricular dysfunction and remodeling (5). Once developed, TR in turn adds volume load and exacerbates remodeling in the right ventricle, potentially leading to impairment of the filling in the LV (6).

Concomitant tricuspid annuloplasty (TAP) during mitral valve surgery is a treatment of choice for addressing secondary TR and is associated with a reduced rate of tricuspid regurgitation progression, improved right ventricular remodeling, and better functional outcomes, without increasing operative mortality (7–11). Recent studies also documented relationships between concomitant TAP and post-operative relief in pulmonary artery hypertension (PH) (12, 13). Most of these evidences are based on the surgery for patients with degenerative mitral valve disease and thus relatively preserved LV systolic function. On the other hand, data regarding the impacts of concomitant TAP on postoperative clinical outcomes in the settings of ventricular dysfunction is still lacking. In patients with ischemic cardiomyopathy, intrinsic ventricular dysfunction as well as the left-sided valve disease affects the pathological pathway of secondary TR development. If left untreated at the timing of mitral surgery, secondary TR can progress and potentially leads to high-risk re-operative tricuspid surgery (14, 15).

We hypothesized that in patients with ischemic cardiomyopathy undergoing restrictive mitral annuloplasty (RMA), concomitant TAP provided a better control of postoperative TR grade and was associated with corresponding relief in PH, as compared with RMA alone. To test our hypothesis, we reviewed the impacts of concomitant TAP on clinical outcomes in patients with ischemic cardiomyopathy undergoing RMA.

## Materials and methods

### Patients

This study included 234 patients with ischemic cardiomyopathy [LV ejection fraction (LVEF) ≤ 40%] who underwent RMA with (*n* = 114) or without (*n* = 120) TAP from 1999 to 2015. All the patients had LV systolic dysfunction and dilation caused by ischemia and significant MR caused by restricted leaflet closure. Indications for tricuspid surgery included moderate or greater degree of TR at patients' maximum symptom and mild degree with significant tricuspid annulus dilation, possibly decided with each surgeons' preference. Patients with degenerative mitral disease and those who underwent surgical ventricular restoration were excluded from this study. A ﬂow diagram depicting the selection of the patients is illustrated in Supplementary Figure S1. Patients data were retrospectively retrieved from the Osaka Cardiovascular Research Group (OSCAR) database, which was approved by the institutional ethics committee of Osaka University Hospital, and written informed consent from each patient was waived for this retrospective study.

## Methods

### Echocardiography

Two-dimensional and Doppler echocardiography procedures were performed both prior to surgery (baseline), and pre-discharge for all patients, of whom 72% (*n* = 169) also had post-discharge study, to assess changes in LV function parameters, left atrial dimension, MR and TR severity and systolic pulmonary artery (PA) pressure. The severity of MR and TR were graded as 0 (absent), 1+ (trivial), 2+ (mild), 3+ (moderate), or 4+ (severe) on the basis of color Doppler extent and spatial distribution of the regurgitant jet relative to the corresponding left and right atrial area.

### Surgical procedures

The surgical procedures were performed with the use of conventional cardiopulmonary bypass with mild hypothermia. Myocardial protection was achieved by both antegrade and retrograde cold blood cardioplegia. All patients underwent stringent restrictive mitral annuloplasty after careful assessments of the inter-commissural distance and height of the anterior leaflet. No other adjunct procedures were performed on the mitral valve itself. For those undergoing tricuspid annuloplasty, prosthetic ring was selected according to the combined surface area of the posterior and anterior tricuspid leaflets, however the ﬁnal decision was made at the discretion of the attending surgeon. Rings were implanted with a standard manner sparing the septal annulus and conduction tissue in the region of the apex of the triangle of Koch.

### Follow-up and assessment of adverse events

After surgery, the patients were kept on standard heart failure medications, including angiotensin-converting enzyme inhibitors or angiotensin-II receptor blockers, β-blockers, and diuretics. The primary endpoint of the study was post-operative recurrent TR, designated for each patient by the earliest echocardiogram on which moderate or greater TR or more than 2 grades of TR progression from preoperative baseline was indicated. The secondly endpoints were postoperative systolic PA pressure, functional changes in LV dimensions and systolic functions, freedom from recurrent MR, defined as moderate or greater MR, cumulative survival, and freedom from death and heart failure readmission. Follow-up was completed in all patients (100%) through a review of their clinical records for total duration of 4.8 ± 3.3 years.

### Statistical analysis

The quantitative data were tested for normality with the Shapiro–Wilk test and presented as mean ± standard deviation or median with interquartile range (IQR) as appropriate. Normally distributed variables were compared with the Student's *t*-test, whereas the Wilcoxon rank-sum test was used for non-normal variables for the two study groups (RMA with vs. without TAP). Pre- and postoperative echocardiographic parameters were compared with paired *t*-test. Categorical variables are shown as frequencies with proportions, and were compared using chi-square analysis or Fisher's exact test, as appropriate.

Calculation of freedom from recurrent TR and recurrent MR, cumulative survival, and freedom from death and heart failure readmission were performed by the Kaplan–Meier method and log-rank testing to compare the groups. Univariable and multivariable Cox proportional hazards regression analyses were performed to determine the association of patients' perioperative characteristics and postoperative recurrent TR. The multivariable model was analyzed with variables with a probability value of less than 0.1 in univariable analysis. The results are summarized as hazard ratios (HRs), 95% conﬁdence intervals (CIs), and *P* values. Statistical significance was determined as *P* < 0.05. The JMP (Version 13; SAS institute Inc, Cary, NC) software was used for statistical analysis.

## Results

### Patients

The baseline and operative characteristics are summarized in Table 1. Preoperatively, there were no intergroup differences in the age, sex, New York Heart Association (NYHA) functional class, and prevalence of chronic kidney disease. However, patients who underwent RMA with TAP tended to present higher prevalence of atrial fibrillation and history of open-heart surgery which accounted for being placed at higher surgical risk as indicated by the higher EuroSCORE II. As for the mitral ring size, the RMA with TAP group tend to receive smaller ring than the RMA alone group (mean ring size 25.2 mm vs. 26.3 mm, *P* < 0.001). The RMA with TAP group received tricuspid ring sizes ranging from 26 to 30 mm; 96% of whom received a ring size of 28 mm or smaller and 95% a semi-rigid prosthetic ring. As a concomitant surgery, atrial fibrillation surgery was more frequently performed, whereas coronary artery bypass grafting was less frequently performed in the RMA with TAP group.

**Table 1:** Patient characteristics and surgical data.

Variables	RMA with TAP (*n* = 114)	RMA alone (*n* = 120)	*P* value
Clinical data			
Age, years	66 ± 9	67 ± 10	0.452
Female sex	24 (21%)	18 (15%)	0.228
Body surface area, m^2^	1.6 ± 0.2	1.7 ± 0.2	0.103
Medical history and presentation at baseline			
NYHA functional class III or IV	84 (74%)	77 (64%)	0.115
EuroSCORE II	9.3 [5.8–16.2]	7.2 [4.4–11.8]	0.016
Chronic kidney disease stage 4 or 5	29 (25%)	31 (26%)	0.945
Atrial fibrillation	24 (21%)	14 (12%)	0.051
Triple-vessel disease	45 (39%)	75 (63%)	<.001
Redo surgery	15 (13%)	4 (3%)	0.005
Echocardiographic data			
Mitral regurgitation grade			
Severe	53 (46%)	27 (23%)	<.001
Tricuspid regurgitation grade			<.001
None or trace	12 (11%)	71 (59%)	
Mild	52 (46%)	45 (38%)	
Moderate	41 (36%)	4 (3%)	
Severe	9 (8%)	0 (0%)	
Pulmonary artery systolic pressure, mmHg	51 ± 16	44 ± 12	<.001
Surgical data			
Mitral ring size			<.001
24 mm	62 (54%)	23 (19%)	
26 mm	39 (34%)	60 (50%)	
28 mm	12 (11%)	32 (27%)	
30 mm or greater	1 (1%)	5 (4%)	
Mitral ring type			0.781
Rigid ring	14 (12%)	11 (9%)	
Semi-rigid ring	94 (83%)	107 (89%)	
Flexible band	6 (5%)	2 (2%)	
Tricuspid ring size			
26 mm	45 (39%)		
27 mm	4 (4%)		
28 mm	60 (53%)		
29 mm or greater	5 (4%)		
Tricuspid ring type			
Semi-rigid ring			
Edwards—MC3	80 (70%)		
Edwards—Cosgrove	28 (25%)		
Flexible band			
St Jude Medical—Tailor	6 (5%)		
Concomitant procedures			
Coronary artery bypass grafting	78 (68%)	97 (80%)	0.03
Arrhythmia surgery	22 (19%)	7 (6%)	0.0014

Values are presented as mean ± standard deviation or number (percentage) as shown. NYHA, New York heart association; RMA, restrictive mitral annuloplasty; TAP, tricuspid annuloplasty.

### Postoperative recurrence or progression of TR

**Figure 1:**
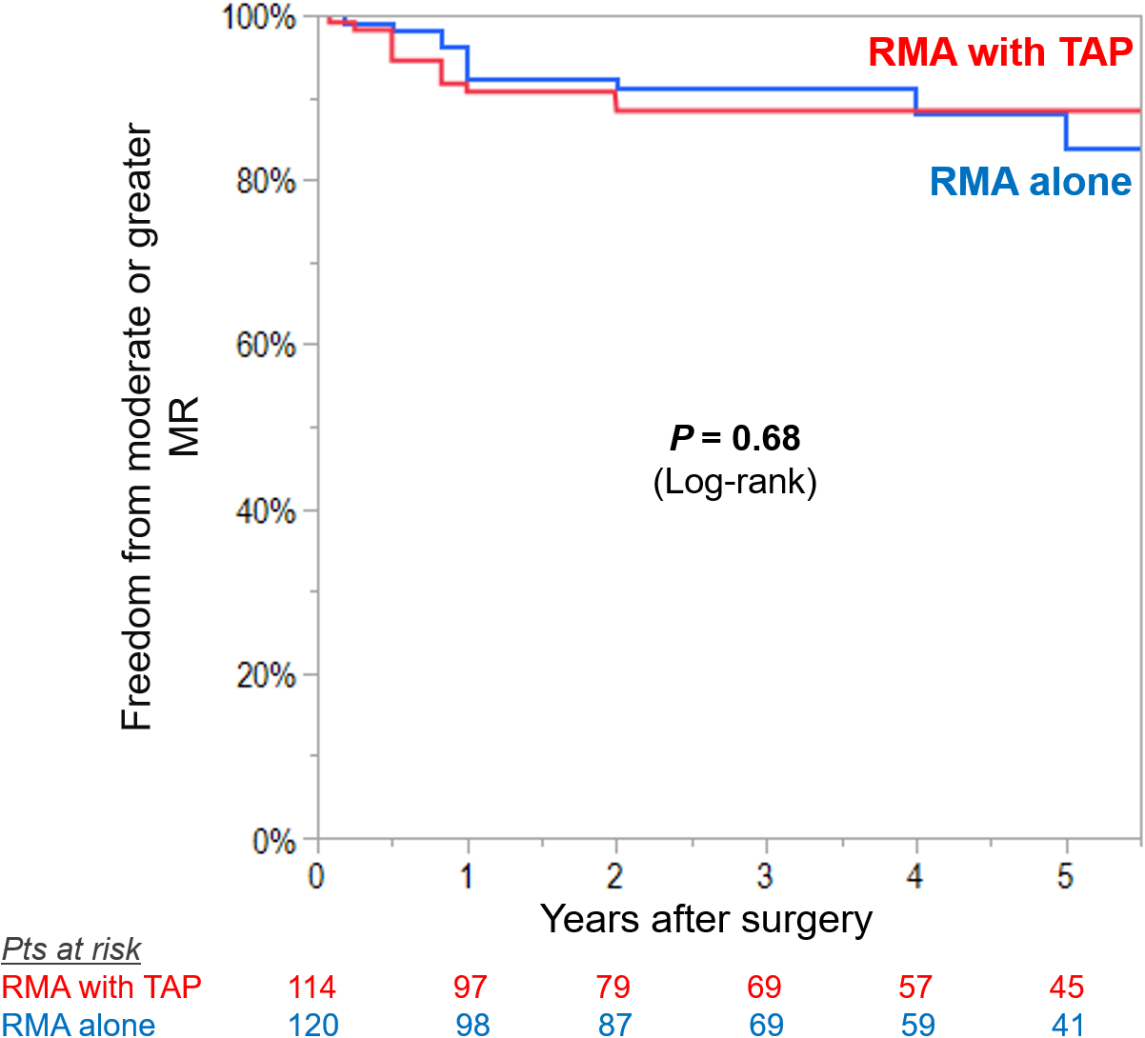
Kaplan–Meier curves for freedom from significant recurrence (moderate or greater) or progression (≥2+ grades from preoperative baseline) in tricuspid regurgitation (TR) in patients undergoing restrictive mitral annuloplasty (RMA) with or without tricuspid annuloplasty (TAP).

At preoperative baseline, RMA with TAP group had significantly advanced degree of TR than did RMA alone group (mean TR grade 2.4 ± 0.8 vs. 1.4 ± 0.6, *P* < 0.001). Freedom from moderate or greater TR and ≥+2 progression at 5 years was 91 ± 3% in RMA with TAP group compared with 81 ± 4% in RMA alone group (log-rank *P* = 0.036) (Figure 1). Multivariable Cox proportion hazards regression analysis identified that TAP procedure was independently associated with freedom from moderate or greater TR and ≥+2 progression of TR (HR: 0.44; 95% CI: 0.20–0.97; *P*-value: 0.034) (Table 2). In subgroup analysis of RMA with TAP group, TAP with flexible band was independent predictor of post-operative recurrence or progression of TR (HR: 6.20; 95% CI: 1.28–30.1; *P*-value: 0.024), whereas in RMA alone group, preoperative mild or greater TR showed a trend for progression of TR (HR: 2.52; 95% CI: 0.99–6.42; *P*-value: 0.052) (Table 3).

**Table 2:** Univariable and multivariable Cox hazards regression analysis for recurrence or progression of tricuspid regurgitation.

Variables	Univariable		Multivariable		
	Hazard ratio	*P* value	Hazard ratio	95% CI	*P* value
Age	1.02	0.21			
Female sex	1.56	0.33			
Body surface area	0.21	0.14			
NYHA functional class III or IV	1.25	0.59			
Chronic kidney disease stage 4 or 5	2.43	0.024	2.46	1.17–5.17	0.023
Atrial fibrillation	0.56	0.31			
Triple-vessel disease	1.08	0.84			
Severe mitral regurgitation at baseline	1.34	0.44			
Pulmonary artery systolic pressure	1.00	0.96			
LV end-systolic dimension	0.96	0.10			
LV ejection fraction	1.03	0.26			
Mitral ring size	1.02	0.88			
Tricuspid annuloplasty	0.45	0.036	0.44	0.20–0.97	0.034

CI, confidence interval; LV, left ventricle; NYHA, New York heart association.

**Table 3:** Univariable and multivariable Cox hazards regression analysis for recurrence or progression of tricuspid regurgitation in patients undergoing restrictive mitral annuloplasty (RMA) with or without tricuspid annuloplasty (TAP).

Variables	Univariable		Multivariable		
	Hazard ratio	*P* value	Hazard ratio	95% CI	*P* value
RMA with TAP group					
Age	0.97	0.38			
Female sex	1.11	0.89			
Body surface area	0.07	0.15			
NYHA functional class III or IV	1.23	0.79			
Chronic kidney disease stage 4 or 5	0.94	0.93			
Atrial fibrillation	0.44	0.38			
Triple-vessel disease	0.19	0.06	0.19	0.02–1.50	0.11
Severe mitral regurgitation at baseline	1.48	0.56			
Pulmonary artery systolic pressure	1.01	0.73			
LV end-systolic dimension	0.94	0.22			
LV ejection fraction	1.02	0.71			
Mitral ring size	1.11	0.65			
Tricuspid annuloplasty with flexible band	6.00	0.03	6.20	1.28–30.1	0.024
RMA alone group					
Age	1.06	0.03	1.04	0.98–1.10	0.20
Female sex	2.09	0.18			
Body surface area	0.24	0.27			
NYHA functional class III or IV	1.49	0.40			
Chronic kidney disease stage 4 or 5	3.70	0.01	3.42	1.34–8.70	0.01
Atrial fibrillation	0.90	0.89			
Triple-vessel disease	1.54	0.37			
Severe mitral regurgitation at baseline	1.78	0.24			
Mild or greater TR at baseline	2.55	0.04	2.52	0.99–6.42	0.052
Pulmonary artery systolic pressure	1.00	0.79			
LV end-systolic dimension	0.97	0.22			
LV ejection fraction	1.04	0.21			
Mitral ring size	0.85	0.26			

CI, confidence interval; LV, left ventricle; NYHA, New York heart association; TR, tricuspid regurgitation.

### Postoperative changes in PH

**Figure 2:**
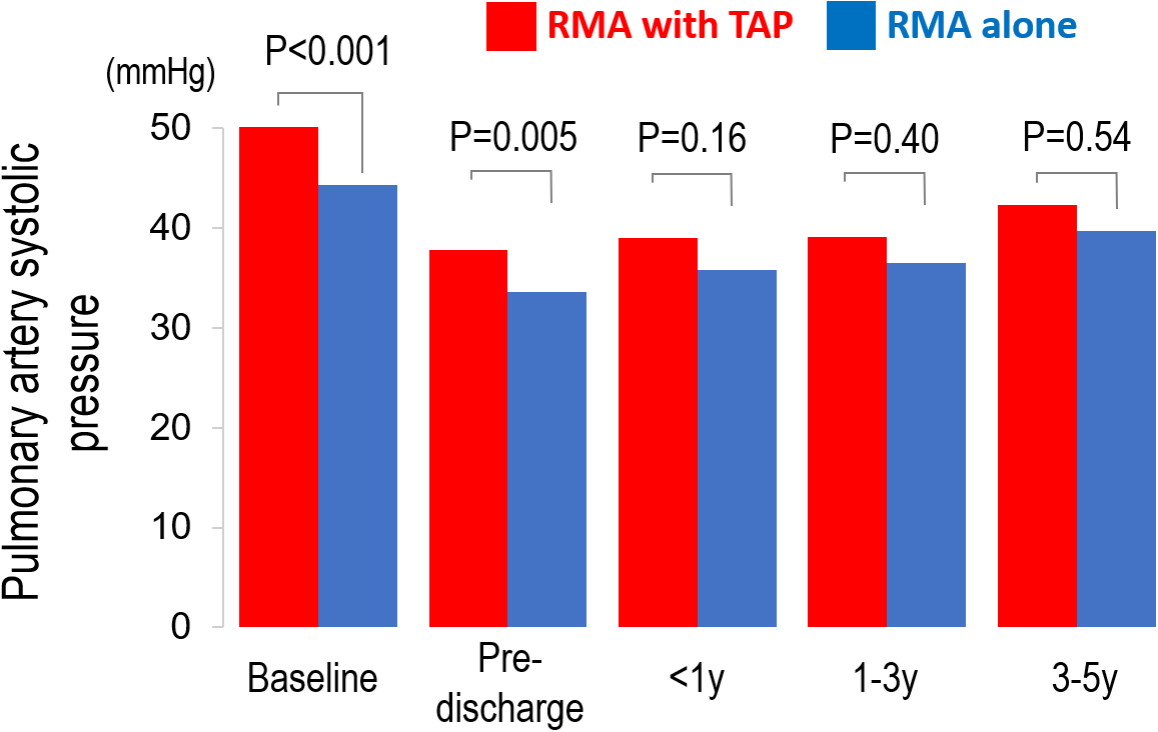
Pre- and post-operative changes in pulmonary artery (PA) systolic pressure in patients undergoing restrictive mitral annuloplasty (RMA) with or without tricuspid annuloplasty (TAP).

At baseline, the RMA with TAP group had significantly higher systolic PA pressure than the RMA alone group (51 ± 16 vs. 44 ± 12 mmHg, *P* < 0.001). After surgery, systolic PA pressure significantly improved in both groups at pre-discharge echocardiography (both *P* < 0.001). At mid-term follow-up, no significant difference in systolic PA pressure between the two treatment groups (RMA with TAP group: 42 ± 18 vs. RMA alone group: 40 ± 16 mmHg *P* = 0.54) (Figure 2).

### Changes in LV function parameters, LA dimension and MR grade

The serial assessments of the echocardiographic parameters according to the study groups are summarized in the Table 4. At baseline, there were no intergroup differences in LV dimensions and ejection fraction, however patients who underwent RMA with TAP tended to show larger LA dimension, as compared with those who underwent RMA alone. Postoperatively, LV dimensions and ejection fraction and LA dimension substantially improved in both groups, yielding comparable values at mid-term follow-up.

**Table 4:** Pre- and postoperative echocardiography.

Variables	Preop	Pre-discharge	<1 year	1–3 year	3–5 year
Echocardiographic parameters					
LV end-diastolic dimension, mm					
RMA with TAP	64 ± 7	57 ± 8	58 ± 8	61 ± 9	61 ± 8
RMA alone	65 ± 7	57 ± 9	58 ± 9	58 ± 9	59 ± 9
*P*-value	0.51	0.97	0.66	0.10	0.58
LV end-systolic dimension, mm					
RMA with TAP	54 ± 7	48 ± 9	47 ± 10	48 ± 11	49 ± 11
RMA alone	55 ± 9	48 ± 9	47 ± 10	46 ± 10	48 ± 11
*P*-value	0.44	0.86	0.88	0.32	0.80
LV ejection fraction, %					
RMA with TAP	30 ± 8	36 ± 11	39 ± 14	40 ± 14	42 ± 14
RMA alone	29 ± 8	34 ± 11	38 ± 12	40 ± 14	38 ± 11
*P*-value	0.29	0.10	0.51	0.75	0.33
Left atrial dimension, mm					
RMA with TAP	48 ± 7	44 ± 7	46 ± 7	47 ± 6	48 ± 6
RMA alone	45 ± 8	42 ± 7	44 ± 8	45 ± 8	45 ± 7
*P*-value	0.012	0.033	0.29	0.25	0.11

Values are presented as mean ± standard deviation or number (percentage) as shown. LV, left ventricle; RMA, restrictive mitral annuloplasty; TAP, tricuspid annuloplasty

**Figure 3:**
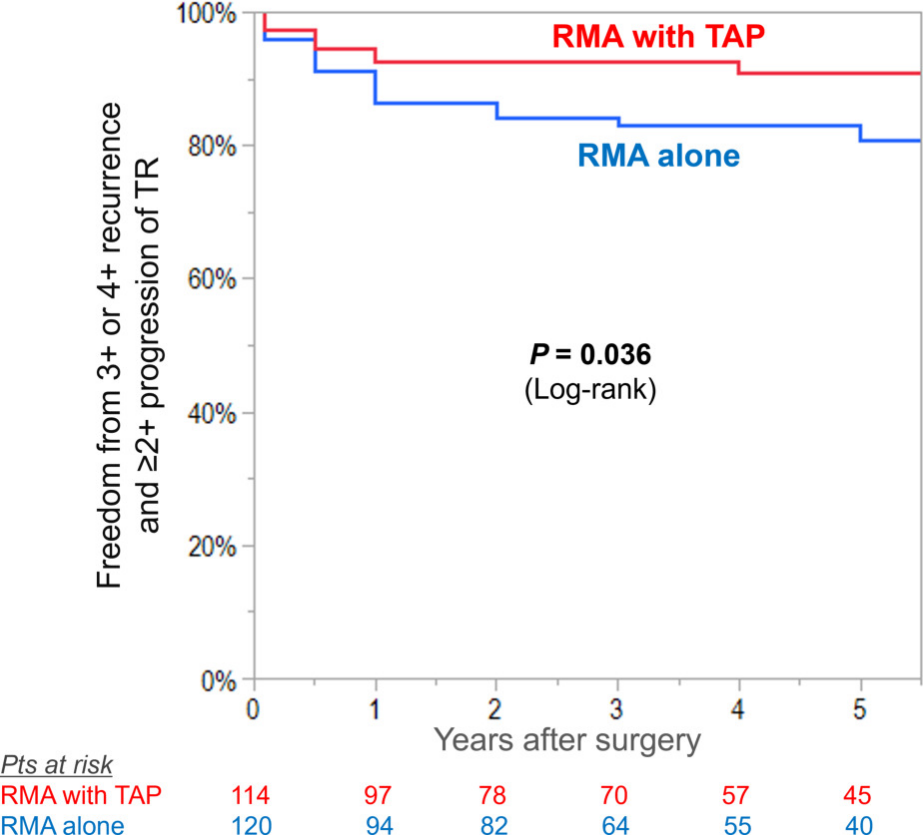
Kaplan–meier curves for freedom from significant recurrence (moderate or greater) in mitral regurgitation (MR) in patients undergoing restrictive mitral annuloplasty (RMA) with or without tricuspid annuloplasty (TAP).

Preoperatively, mean MR grade was higher in RMA with TAP group compared with RMA alone group (mean MR grade 3.3 ± 0.7 vs. 3.0 ± 0.7, *P* < 0.001). Freedom from moderate or greater MR was not different between the groups (RMA with TAP vs. RMA alone, 88 ± 3% vs. 84 ± 4%, log-rank *P* = 0.68) (Figure 3).

### Early and long-term clinical outcomes

**Figure 4:**
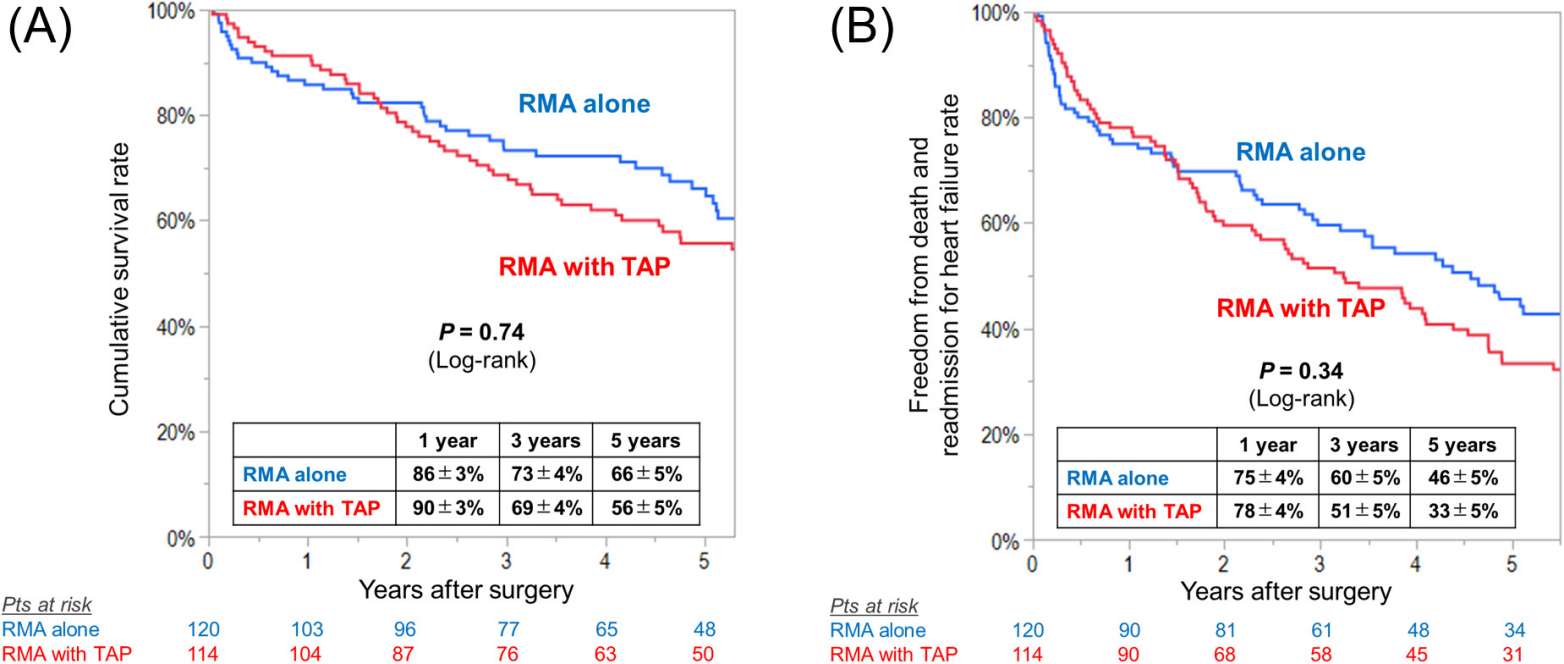
Kaplan–meier curves for **(A)** overall survival and **(B)** freedom from death and readmission for heart failure in patients undergoing restrictive mitral annuloplasty (RMA) with or without tricuspid annuloplasty (TAP).

There were no inter-group differences in the rates of 30-day mortality [RMA with TAP vs. RMA alone, 0.9% (*n* = 1) vs. 0.8% (*n* = 1), *P* = 0.97] and hospital mortality [9% (*n* = 10) vs. 8% (*n* = 10), *P* = 0.90]. During follow-up, 54 (47%) and 68 (57%) died in the RMA with TAP and in the RMA alone group, respectively, and the cumulative survival rates at 1, 3, and 5 years was 90%, 69% and 56% and 86%, 73%, and 66%, respectively (log-rank *P* = 0.74) (Figure 4A). The rates of freedom from death and readmission for heart failure were not also different between the groups (log-rank *P* = 0.34) (Figure 4B).

## Discussion

The major findings of this study can be summarized as follows. In a specific cohort of patients with ischemic cardiomyopathy undergoing RMA, (i) patients who were indicated for concomitant TAP procedure showed severer TR and higher systolic PA pressure at baseline, in along with more severe MR grade, (ii) though, they achieved greater freedom from recurrence or progression of TR compared with those received RMA alone, yielding nearly identical systolic PA pressure at mid-term follow-up, and (iii) no significant between-group difference was demonstrated in early and long-term survival, despite unfavorable baseline characteristics for RMA with TAP group. Notably, we found that concomitant TAP procedure was independently associated with greater freedom from postoperative TR, an independent predictor of mortality in patients with impaired LV dysfunction secondary to ischemic insult. In addition, at mid-term follow up, RMA with TAP group demonstrated nearly identical systolic PA pressure despite preoperative higher values compared with RMA alone group.

One of the important findings of this study was to delineate the independent relationship between concomitant TAP and postoperative TR reduction in ischemic cardiomyopathy. In patients with reduced LV function, secondary TR is associated with poor prognosis and functional status even though mitral lesion is addressed (16, 17). In our study, patients undergoing RMA with TAP achieved significantly lower prevalence of recurrence or progression of TR than did those undergoing RMA alone. However, controversies still exist regarding the impact of concomitant TAP on postoperative TR reduction. Navia and colleagues reviewed 568 patients with ischemic MR undergoing mitral surgery with (*n* = 131) or without (*n* = 437) TAP (18). Postoperatively, the TAP group demonstrated less severity in TR grade (the prevalence in ≥mild TR, 62% vs. 77%) than the no-TAP group, despite severer post-operative MR grade (the prevalence in ≥moderate MR, 16% vs. 10%). Whereas another trial reported by Matsunaga and colleagues refuted the benefit of concomitant TAP (19). They included 21 patients with ischemic MR (mean LVEF 42 ± 13%) undergoing RMA with (*n* = 9) or without (*n* = 12) tricuspid valve repair. At the mean echocardiographic follow-up of 35 ± 24 months, the incidence of recurrent TR (≥moderate grade) was not statistically different between the treatment groups (44% vs. 67%). The relatively high incidence of recurrent TR in the TAP group can be partly attributed to the utilization of a de Vega procedure performed in almost half of the patients in the repair group, considering that recurrent TR was observed more frequently after annuloplasty with de Vega procedure than that with prosthetic rings (3). Our data seems to be consistent with the results from Navia and colleagues with regard to the significant reduction in TR grade for the concomitant TAP group, although the prevalence in post-operative recurrent TR seems to be lower in the present series. Multivariate analysis in our study confirmed that concomitant TAP was independently associated with greater freedom from recurrent TR, suggesting potential benefit of the strategy of concomitant TAP for controlling TR in patients with advanced ischemic cardiomyopathy.

Another novel finding of this study was to identify the relationship between concomitant TAP strategy and postoperative reduction in PH, an important risk factor for survival, in patients with end-stage cardiomyopathy (20–22). The association between concomitant TAP strategy and post-operative reduction in PH has been demonstrated for those with degenerative mitral disease with preserved LV function (12, 13). Chen and colleagues analyzed pre- and postoperative changes in systolic PA pressure in 137 patients who underwent TAP with left-side valve surgery (13). The proportion of patients with severe PH significantly reduced from 48% at the baseline to 12% postoperatively. Chikwe and colleagues also reviewed 645 patients undergoing degenerative mitral repair with (*n* = 419) or without (*n* = 226) concomitant TAP (12). Whereas the TAP group had significantly higher systolic PA pressure at baseline (36 vs. 32 mmHg) than those without TAP, they postoperatively achieved nearly identical systolic PA pressure without PH. Our results were compatible with these studies in terms of significant reduction in PH after mitral valve surgery with concomitant TAP, though the postoperative systolic PA pressure was still high in our study (mean PA systolic pressure at mid-term follow-up, RMA with TAP group: 42 ± 18 and RMA alone group: 40 ± 16 mmHg). This difference can be accounted for the fact that our cohort included those with ischemic cardiomyopathy, whose intrinsic ventricular pathology cannot be totally addressed with the surgical procedure for the valve alone. In fact, although RMA procedure can significantly improve MR severity, it did not normalize LV function parameters (i.e., LV dimensions and ejection fraction), but partially reverse LV remodeling or slow down its progression. Possibly, surgical correction of MR cannot completely repair myocardial damage due to ischemic insult or permanently halt LV remodeling processes. Nevertheless, our result supports the idea that concomitant TAP was associated with relieving PH severity to some degree even in patients accompanying ventricular dysfunction.

Both pre-operative significant TR and PH are the powerful predictors of mortality in patients with impaired LV function (17, 21). Therefore, it is reasonable to expect that among our series of patients, those who underwent RMA plus TAP would have a worse prognosis than those who underwent RMA alone, because the former group of patients had a substantially severer TR and PH at baseline (before surgery). In addition, they were considered sicker cohorts at baseline and therefore at high risk for surgery, as evidenced by significantly higher EuroSCORE II. However, contrary to our expectation, concomitant TAP did not increase early mortality rate, supporting the idea that concomitant TAP procedure can be performed with acceptable surgical risk for patients with ischemic cardiomyopathy. Notably, patients who underwent RMA plus TAP achieved comparable long-term survival to those who underwent RMA alone, which was another important finding of this study. So far, scarce data is available regarding the long-term survival after concomitant TAP for those reduced LV function and the impact of concomitant TAP and RMA on long-term survival in patients with advanced cardiomyopathy remains to be determined.

## Limitations

There are some limitations to our study. First, this study was retrospective in nature and included a small number of subjects; thus, our results should be interpreted cautiously until verified in an independent, prospective study. As for the assessment in TR severity and systolic PA pressure, we utilized Doppler-derived parameters obtained by echocardiography, which are not as precise as measurements obtained with a detailed imaging modality such as computed tomography or magnetic resonance imaging or direct measurement by catheter. Impacts of potential confounders such as adjunct coronary artery bypass grafting or Maze procedure on clinical outcomes were not adjusted. Right ventricular function was not assessed. Finally, further investigation of a larger patient population with a longer follow-up is required to definitively confirm our results.

## Conclusions

In patients with ischemic cardiomyopathy undergoing RMA, concomitant TAP procedure was independently associated with greater freedom from post-operative recurrence or progression of TR. Concomitant TAP was performed for those with advanced grade of TR and elevated PA pressure. After surgery, TAP was associated with greater reduction in TR, yielding a comparable PH severity. Whether substantial improvements in TR and PH resulting from RMA and concomitant TAP gives a survival benefit remains to be determined.

## Data Availability

The data analyzed in this study is subject to the following licenses/restrictions: The deidentified participant data will be shared on a request basis. Please directly contact the corresponding author to request data sharing. Requests to access these datasets should be directed to Yusuke Misumi, y-misumi@surg1.med.osaka-u.ac.jp.

## Osaka Cardiovascular Surgery Research (OSCAR) study group

This study was conducted with patient data retrieved from the Osaka Cardiovascular Research Group (OSCAR) database, which was approved by the institutional ethics committee of Osaka University Hospital, and written informed consent from each patient was waived for this retrospective study (Reference no. 08218-6).

## Supplementary material

Supplementary Figure 1 Participant flow chart.
