# Supplementary figures and images for: Tricuspid annuloplasty in ischemic cardiomyopathy patients undergoing restrictive mitral annuloplasty
Source: Front Cardiovasc Med. 2025 May 14;12:1542619. doi: 10.3389/fcvm.2025.1542619 (PMC12116392; doi:10.3389/fcvm.2025.1542619)

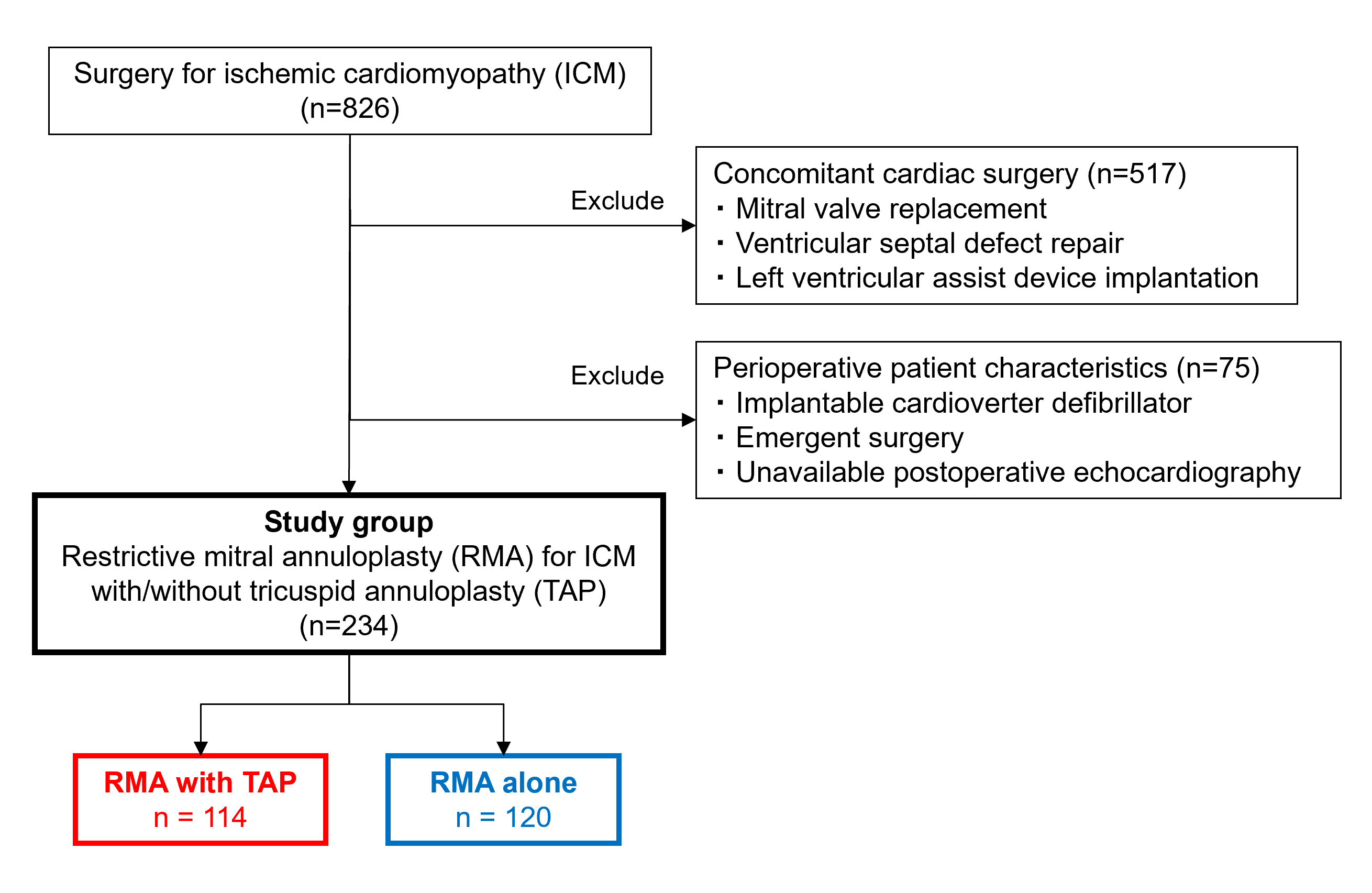

Supplement: Supplementary Figure 1 — Participant flow chart. [file Image1.tif]
